# Tolvaptan use in cancer patients with hyponatremia due to the syndrome of inappropriate antidiuretic hormone: a post hoc analysis of the SALT‐1 and SALT‐2 trials

**DOI:** 10.1002/cam4.805

**Published:** 2017-03-02

**Authors:** Richard J. Gralla, Fatima Ahmad, Jaime D. Blais, Joseph Chiodo, Wen Zhou, Linda A. Glaser, Frank S. Czerwiec

**Affiliations:** ^1^ Albert Einstein College of MedicineBronxNew York; ^2^ Otsuka Pharmaceutical Development & Commercialization Inc.RockvilleMaryland; ^3^ Coastal Biomedical Research, Inc.Santa MonicaCalifornia

**Keywords:** breast cancer, head and neck cancer,, Hyponatremia,, lung cancer,, renal cancer, syndrome of inappropriate antidiuretic hormone (SIADH), tolvaptan

## Abstract

Hyponatremia is a common electrolyte disorder in cancer patients and has been associated with poor prognosis. A frequent cause of cancer‐related hyponatremia is the syndrome of inappropriate antidiuretic hormone (SIADH). This study was a post hoc subgroup analysis of the SALT‐1 (Study of Ascending Levels of Tolvaptan in Hyponatremia) and SALT‐2 clinical trials. Hyponatremic subjects with SIADH and cancer received the oral selective vasopressin V2‐receptor antagonist tolvaptan (*n* = 12) or matching placebo (*n* = 16) once‐daily for 30 days. The initial tolvaptan dose (15 mg) was titrated over 4 days to 30 or 60 mg per day, as needed, according to serum sodium level and tolerability. Baseline serum sodium levels in the SIADH/cancer cohort of the SALT trials was 130 and 128 mEq/L for tolvaptan and placebo, respectively. Mean change from baseline in average daily serum sodium AUC for tolvaptan relative to placebo was 5.0 versus −0.3 mEq/L (*P* < 0.0001) at day 4, and 6.9 versus 1.0 mEq/L (*P* < 0.0001) at day 30; the observed treatment effects were similar to those in the overall SIADH population (i.e., with and without cancer) at both time points. Serum sodium normalization was observed in 6/12 and 0/13 subjects at day 4 and 7/8 and 2/6 subjects at day 30 in the tolvaptan and placebo groups, respectively (*P* < 0.05 for both). Common treatment‐emergent AEs for tolvaptan were consistent with previously reported results. In this post hoc study of the SALT trial population, oral tolvaptan was an effective and safe therapy for the treatment of hyponatremia in subjects with SIADH and cancer.

## Introduction

Hyponatremia (serum sodium <135 mEq/L) is a common electrolyte disorder in patients with cancer 1. In one study, approximately 14% of medical inpatients with a diagnosis of low serum sodium also had a tumor‐related condition 2. Particularly frequent in small‐cell lung cancer (SCLC), where it has been reported to affect 25–44% of patients 3, 4, 5, 6, hyponatremia is also commonly found in head and neck cancer and breast cancer and can be a complicating factor in a wide array of other malignancies 1, 7. Cancer‐related hyponatremia correlates with poor outcome, including significantly shorter survival in studies in patients with SCLC 7. Moreover, in one SCLC study, patients whose serum sodium was normalized survived longer than those who remained hyponatremic 6. Although not studied as extensively in other cancers, hyponatremia has also been found to be associated with poor prognosis in non‐small‐cell lung cancer 8, 9, 10, renal cell carcinoma 11, 12, gastric cancer^13^, ^14^, and non‐Hodgkin's lymphoma 15.

Cancer‐related hyponatremia occurs by multiple mechanisms involving both inherent pathophysiologic processes and iatrogenic causes 1, 7. One of the most common mechanisms is the syndrome of inappropriate antidiuretic hormone (SIADH), which occurs in 11–15% of patients with SCLC 16, 17 and 3% of patients with head and neck cancer 18. Para‐neoplastic SIADH is thought to be caused primarily by the ectopic secretion from tumor cells of arginine vasopressin (AVP; also known as antidiuretic hormone [ADH]) 7. Under normal conditions, AVP is secreted by the posterior pituitary in response to increases in plasma osmolality or reductions in blood volume or pressure 19. AVP activates V2‐receptors in the renal collecting duct to promote free water absorption, thereby increasing extracellular fluid volume 20. The persistent unregulated expression of AVP in para‐neoplastic SIADH leads to excessive dilution of free sodium, the primary etiology of the observed hyponatremia. It should be noted that other etiologies of hyponatremia have been described in cancer patients 1. Moreover, many anticancer drugs, including cyclophosphamide, vinca alkaloids, platinum compounds and alkylating agents, as well as multiple palliative medications, have been implicated in the development of hyponatremia in some cancers 7.

Tolvaptan (SAMSCA^®^, Otsuka America Pharmaceutical, Inc., Rockville, MD) is an oral selective V2‐receptor antagonist indicated for the treatment of clinically significant hypervolemic and euvolemic hyponatremia 21. In a subanalysis of the Study of Ascending Levels of Tolvaptan in Hyponatremia (SALT) clinical trials 22, tolvaptan therapy resulted in a statistically significant (*P* < 0.0001) serum sodium correction relative to placebo in a general population of SIADH subjects, that is, one that included subjects both with and without cancer 23. Tolvaptan provided a significant positive treatment effect on the mental component summary, while there was no change on the physical component summary scores of the SF‐12 Health Survey. Additionally, there was a trend toward reduction in the need for fluid restriction (*P* = 0.08). The most common treatment‐related adverse events were related to the free water clearance effects, including thirst and dry mouth.

Tolvaptan presents a treatment option that is easy to employ for health care professionals and patients in that it is a daily oral agent that can eliminate the need for fluid restriction and/or electrolyte infusions. Based on these factors, as well as the prior positive results in a general population of subjects with hyponatremia and SIADH, we performed a further subanalysis to assess the activity and tolerability of tolvaptan in those subjects from the SALT clinical trials who had hyponatremia associated with SIADH and cancer.

## Materials and Methods

### Study design

SALT‐1 and SALT‐2 (clinicaltrials.gov identifiers: NCT00072683 and NCT00201994) were nearly identical phase 3, multicenter, double‐blind, placebo‐controlled trials assessing the efficacy and safety of tolvaptan in subjects with euvolemic or hypervolemic hyponatremia 22. Adults 18 years of age or older with baseline serum sodium concentrations <135 mEq/L in association with chronic heart failure, cirrhosis or SIADH were randomized to once‐daily tolvaptan (*n* = 225) or matching placebo (*n* = 223) for 30 days. The initial dose of tolvaptan, 15 mg per day, was titrated to 30 or 60 mg per day if the patient's serum sodium remained below 136 mEq/L and had increased by less than 5 mEq/L over the prior 24 h 22. The option of decreasing study medication dosage during the treatment period was available at the discretion of the investigator. Standard of care therapies for hyponatremia were also permitted at the investigator's discretion, including fluid restriction (1 L/day or less of fluids) in subjects with serum sodium < 130 mEq/L, although withholding liquids was discouraged during the first 24 h in order to determine the rate and magnitude of serum sodium change on study drug. Demeclocycline, lithium chloride, and urea were not allowed.

A total of 206 of 448 subjects in the SALT‐1 and SALT‐2 trials had “SIADH” or “other” identified as the primary etiology of their hyponatremia (188 were euvolemic and 18 were hypervolemic). Subjects in the “other” group could not be allocated to any of the three primary hyponatremia etiology groups (chronic heart failure, cirrhosis, or SIADH). Of the 206 subjects, 110 had “investigator‐diagnosed SIADH” (58 in the tolvaptan group and 52 in the placebo group) and 96 had an etiology of “other.”^23^ This study is a post hoc, exploratory subanalysis of the SALT trials focusing exclusively on participants with a primary etiology of SIADH/other and who also had a diagnosis of cancer. For comparative purposes, outcomes in the SIADH/cancer subgroup are presented relative to the overall population with hyponatremia attributable to SIADH (or other; *n* = 5) from the SALT studies.

### Assessments

Study assessments occurred at baseline, 8 h after the first administration of study drug, and 2, 3, 4, 11, 18, 25, and 30 days. The coprimary endpoints of the SALT trials, also assessed in this subanalysis, were the average area under the serum sodium concentration curve (AUC) from baseline to day 4 and from baseline to day 30. Secondary endpoints in the SALT trials examined in this subanalysis included: the mean change from baseline in serum sodium concentration at 8 h and on day 4 and 30; the percentage of subjects with normalized serum sodium at 8 h and on day 4 and day 30; and the percentage of subjects who required fluid restriction at any time on therapy. Safety was assessed by adverse events and included analysis of the seriousness and severity of each event, as well as the relationship of each event to study treatment.

### Statistics

All reported *P*‐values are two‐sided. The change in the average daily AUC for the serum sodium concentration from baseline to day 4 and from baseline to day 30 was calculated as the AUC for each patient, divided by the observation period (4 or 30 days), minus the baseline value. The serum sodium concentration changes in the two study groups were compared with an analysis of covariance (ANCOVA) model in which the group assignment and baseline stratification factors were covariates. Serum sodium concentration changes were compared between study groups with the use of the ANCOVA model and the covariates noted above. The percentage of subjects normalizing serum sodium concentration (>135 mEq/L) or requiring fluid restriction was analyzed with the Cochran–Mantel–Haenszel test, stratified by baseline disease severity and origin. The results of this study were not adjusted for multiplicity and should be interpreted as hypothesis generating.

### Ethics

SALT‐1 and SALT‐2 were conducted in accordance with the Declaration of Helsinki. Each study followed a protocol reviewed by an institutional review board or ethics committee, and all subjects provided written informed consent prior to receiving study treatments.

## Results

### Patients

**Figure 1:**
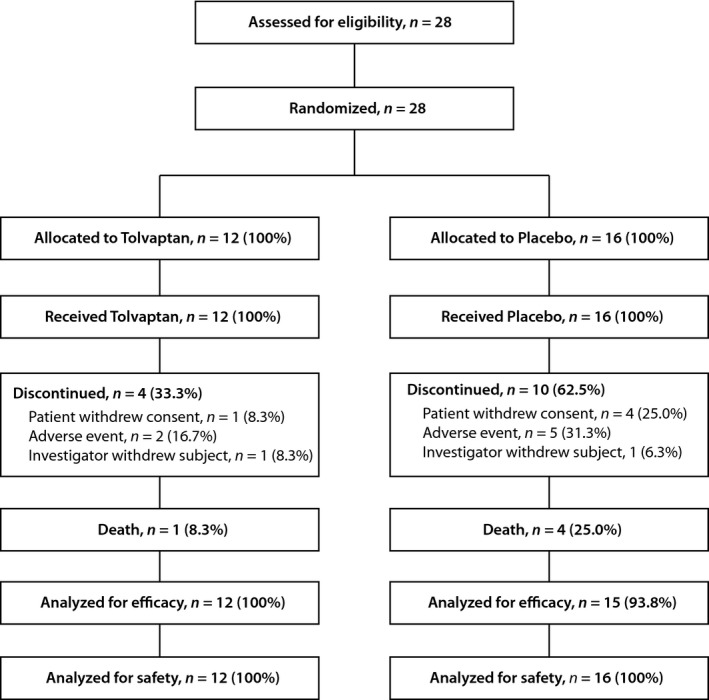
Consort flow diagram. Eligible subjects were from the SALT study population and included those who had hyponatremia in association with SIADH (or other etiology besides congestive heart failure or liver cirrhosis) 22. SALT, Study of Ascending Levels of Tolvaptan in Hyponatremia; SIADH, syndrome of inappropriate antidiuretic hormone.

A total of 28 subjects in the SALT‐1 and SALT‐2 trials had cancer and SIADH, 12 of whom were randomized to the tolvaptan arm and 16 to the placebo arm (Fig. 1). The most common cancers in the SIADH/cancer subgroup were lung (29%), head and neck (25%), breast (11%), and renal (11%). All subjects in both treatment groups were available for safety analysis. All but one of the 28 subjects (96%) were available for the assessment of efficacy. Two subjects in the tolvaptan arm discontinued before completion (one patient died during the evaluation), and four subjects on the placebo arm died during the course of the study. The two treatment groups were well matched for age and baseline serum sodium concentration (130 ± 2.9 vs. 128 ± 5.2 mEq/L); however, they differed in gender frequency (male, 33% vs. 62.5%) and cancer subtype distribution (Table 1).

**Table 1:** Demographics and baseline characteristics

	Tolvaptan	Placebo
*N*	12	16
Age, mean years ± SD	63 ± 12	65 ± 10
Male, %	33.3	62.5
Caucasian, %	75.0	100.0
Baseline serum sodium, mean mEq/L ± SD	130 ± 2.9	128 ± 5.2
Tumor types, *n*		
Lung	1	7
Head and neck	4	3
Breast	2	1
Renal	1	3
Ovarian	1	0
Gall bladder	1	0
Skin	1	0
Uterine	1	0
Prostate	0	1
Adenocarcinoma of the mediastinum	0	1

### Efficacy

**Figure 2:**
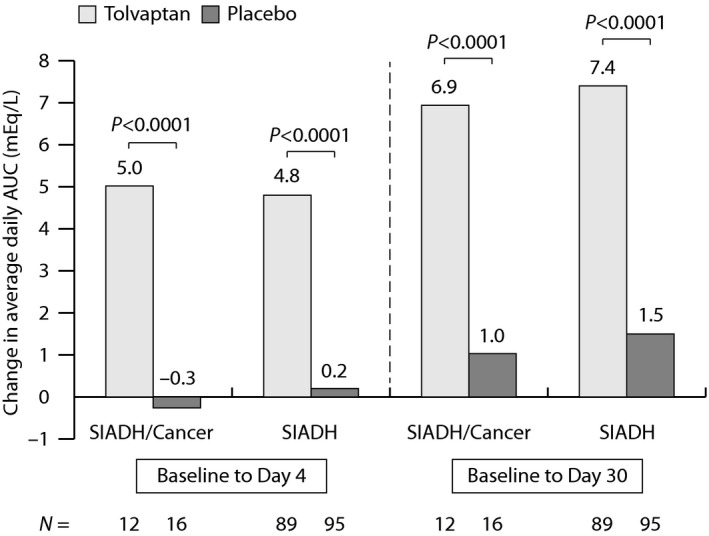
Mean change from baseline in daily serum sodium AUC at day 4 and day 30 in the SIADH/cancer subgroup and the overall SIADH population of SALT. Values based on the intent‐to‐treat population (all randomized subjects treated with study medication who had a baseline serum sodium observation and at least one postbaseline observation within the trial treatment period or no more than 1 day after the day of the last dose). *P*‐values were derived from an ANCOVA model with factors of trial, treatment, baseline hyponatremia severity, and baseline serum sodium concentration as covariates. SALT, study of ascending levels of tolvaptan in hyponatremia; SIADH, syndrome of inappropriate antidiuretic hormone.

**Figure 3:**
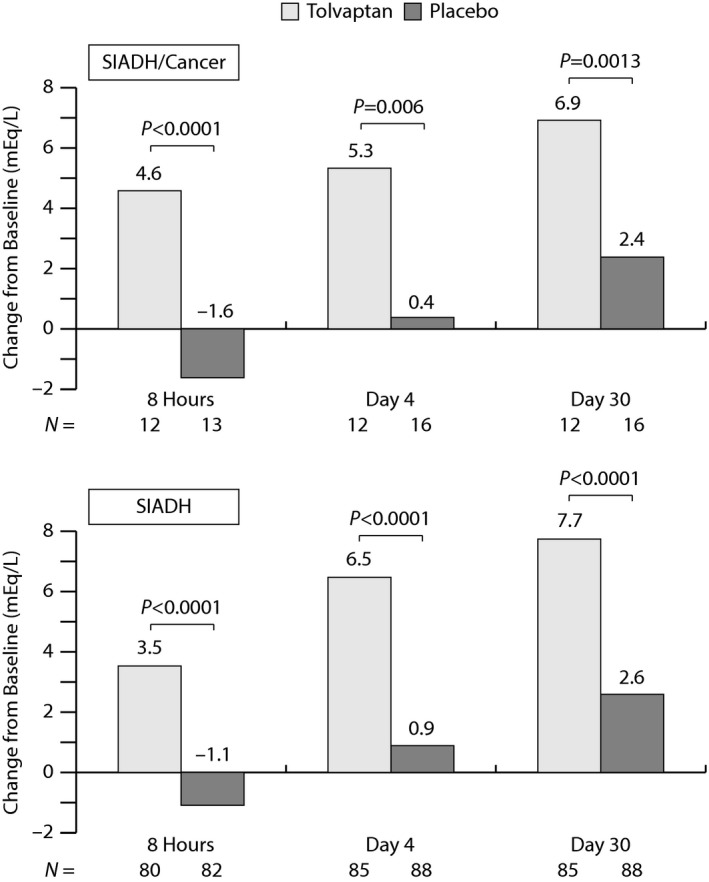
Mean change from baseline in serum sodium levels by visit in the SIADH/cancer subgroup (top) and the overall SIADH population (bottom) of SALT. Missing data were imputed by the last‐observation‐carried‐forward methodology. *P*‐values were derived from an ANCOVA model, with factors of treatment, baseline hyponatremia severity and baseline serum sodium level, and origin as covariate. SALT, study of ascending levels of tolvaptan in hyponatremia; SIADH, syndrome of inappropriate antidiuretic hormone.

The primary efficacy outcome in the SALT trials was the change in average daily serum sodium AUC from baseline to day 4 and from baseline to day 30. For the SIADH/cancer subgroup, subjects on tolvaptan exhibited a highly significant improvement in this parameter (*P* < 0.0001) relative to placebo to day 4 (5.0 vs. −0.3 mEq/mL) and to day 30 (6.9 vs. 1.0 mEq/L) (Fig. 2). The responses in the SIADH/cancer subgroup were consistent with those in the overall SIADH population. Mean change from baseline in serum sodium concentrations were significantly higher in the tolvaptan group compared to the placebo group at 8 h and at all subsequent study visits. Again, the effects in both treatment groups were similar to those observed in the overall SIADH population (Fig. 3).

**Figure 4:**
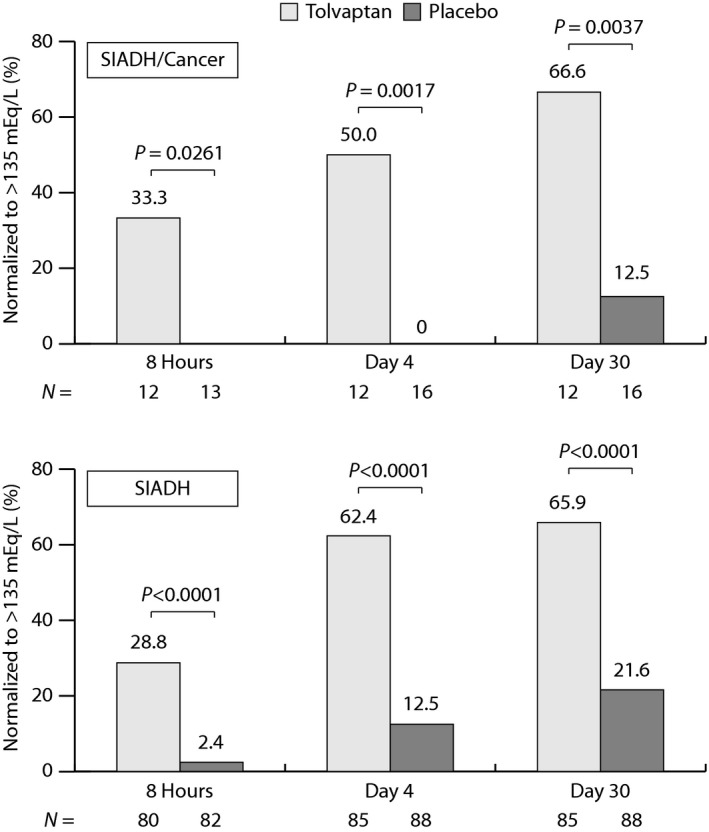
Percentage of subjects normalizing serum sodium levels to >135 mEq/L in the SIADH/cancer subgroup (top) and the overall SIADH population (bottom) of SALT. All randomized subjects with postbaseline serum sodium observations were included in the analysis. Missing data were imputed by the last‐observation‐carried‐forward methodology. *P* values were derived from the Cochran–Mantel–Haenszel test, stratified by baseline disease severity and origin. SALT, study of ascending levels of tolvaptan in hyponatremia; SIADH, syndrome of inappropriate antidiuretic hormone.

In the SIADH/cancer subgroup, significantly higher percentages of subjects normalized their serum sodium (>135 mmEq/L) on tolvaptan relative to placebo at each tested time point: 33.3% versus 0% (*P* = 0.0261) at 8 h, 50.0% versus 0% (*P* = 0.0017) at day 4, and 66.6% versus 12.5% (*P* = 0.0037) at day 30 (Fig. 4). These findings were similar to those reported for the overall SIADH population.

In total, 8.3% and 18.7% of SIADH/cancer subjects required fluid restriction in the tolvaptan and placebo treatment groups, respectively, compared with 7.8% and 13.7% in the overall SIADH population. Differences in rates of fluid restriction between treatment groups were statistically significant for the overall SIADH population (*P* < 0.05), but were not statistically significant in the smaller SIADH/cancer subgroup.

### Safety

Adverse events related to study treatment in the SIADH/cancer population were consistent with previously reported results (Table 2). With the exception of one case of severe nausea, all adverse events in the tolvaptan group were mild to moderate in intensity. No patient exhibited overly rapid correction of serum sodium, defined as >8 mEq/L change by first 8 h or >12 mEq/L change by the first 24 h.

**Table 2:** Adverse events occurring with incidence >10% in either treatment group

	Tolvaptan (*N* = 12) *n* (%)	Placebo (*N* = 16) *n* (%)
Fatigue	3 (25.0)	0 (0.0)
Anemia	2 (16.7)	1 (6.3)
Abdominal pain	2 (16.7)	1 (6.3)
Constipation	2 (16.7)	1 (6.3)
Diarrhea	2 (16.7)	1 (6.3)
Headache	2 (16.7)	1 (6.3)
Nausea	2 (16.7)	0 (0.0)
Hypokalemia	2 (16.7)	0 (0.0)
Arthralgia	2 (16.7)	0 (0.0)
Dry mouth	1 (8.3)	2 (12.5)
Dizziness	1 (8.3)	2 (12.5)
Tremor	1 (8.3)	2 (12.5)
Pyrexia	0 (0.0)	2 (12.5)
Thirst	0 (0.0)	2 (12.5)
Hematocrit decreased	0 (0.0)	2 (12.5)
Hemoglobin decreased	0 (0.0)	2 (12.5)
Anxiety	0 (0.0)	2 (12.5)

## Discussion

In this subanalysis of the SALT‐1 and SALT‐2 clinical trials, oral tolvaptan significantly improved serum sodium levels relative to placebo in hyponatremic SIADH subjects with cancer. Consistent results were seen for all endpoints, including average daily serum sodium AUC, mean change from baseline in serum sodium concentration, and percentage of subjects with normalized sodium. Statistically significant treatment effects were observed as early as 8 h post administration of once‐daily oral tolvaptan. These favorable treatment effects for tolvaptan paralleled those described previously in the overall SIADH population 23. It is worth noting that normalization of sodium levels was sustained over 4 years of tolvaptan therapy (up to 214 weeks) in the SIADH/other group 24. although the effects of long‐term tolvaptan use in the cancer subgroup remains untested. Finally, there was no evidence of cancer‐specific differences in tolvaptan response, although patient numbers were small.

The side effect profile of tolvaptan in subjects with SIADH and cancer was consistent with the profiles observed in the overall SIADH population 23, as well as the complete SALT‐1 and SALT‐2 study population (in addition to cancer subjects, the SALT trials had large numbers of subjects with congestive heart failure and cirrhosis) 22. In both of the prior published analyses, the most common adverse events were related to the mechanism of action of tolvaptan and included thirst (SALT: 14%; SALT/SIADH: 17.6%) and dry mouth (SALT: 13%; SALT/SIADH: 15.7%). Both of these mechanism‐driven events occurred at lower frequencies in the tolvaptan group than the placebo group in the small SIADH/cancer cohort of SALT. Nearly all adverse events in SIADH/cancer subjects on tolvaptan were mild to moderate in intensity, and all adverse events were medically manageable.

The most important safety concern for any hyponatremia treatment is overly rapid serum sodium correction, which can cause osmotic demyelination resulting in dysarthria, mutism, dysphagia, lethargy, affective changes, spastic quadriparesis, seizures, coma, and death. A practical approach was employed in the SALT trials to manage these risks. First, subjects initiated tolvaptan therapy at 15 mg per day. Then, during the initial 4 days of therapy, this starting dose was increased to 30 mg and 60 mg according to a regimen designed for slow correction of serum sodium concentrations. Specifically, if the serum sodium concentration remained below 136 mEq/L at a particular tolvaptan dose and had increased by less than 5 mEq/L during the prior 24 h, the dose was increased one step. If the serum sodium concentration rose above 145 mEq/L or increased at too great a rate (e.g., >12 mEq/L over 24 h or >8 mEq/L over the first 8 h of therapy), then the next dose of tolvaptan was either withheld or decreased, or the patient's fluid intake was increased. By following this relatively straightforward regimen, only four of 223 tolvaptan subjects (1.8%) in the overall SALT study population exceeded the desirable rate of sodium correction (maximum observed rate, 0.61 mEq/L per h; despite the over‐rapid correction, no symptoms of osmotic demyelination syndrome were observed in these 4 subjects) 22. None of the four subjects were in the SIADH/cancer cohort.

The analysis presented here is post hoc in nature, carrying the risk of a false positive result, and should therefore be considered as hypothesis generating. While the observed efficacy and safety results from subjects with cancer and hyponatremia attributed to SIADH were consistent with results seen in the overall SALT‐1 and SALT‐2 trials, this study was not powered to analyze these subgroups independently. Nonetheless, the findings reported here represent one of the largest randomized therapeutic experiences in cancer patients with SIADH. Considering the potential benefits of tolvaptan, including once‐daily administration, relatively quick onset of improvement in hyponatremia, a favorable safety profile, and ease of adherence for patients (unlike with fluid restriction or infusion therapy), the results support the use of tolvaptan in this cancer population. Further studies in subjects with cancer and SIADH, as well as in cancer subjects with hyponatremia of other etiologies, are warranted.

## Conflicts of Interest

Richard J. Gralla has been a consultant to Otsuka Pharmaceuticals. Linda A. Glaser has received clinical research funding from Merck, Takeda, Pfizer, Vertex Pharmaceuticals, Seratrials and Akebia Pharmaceuticals and currently serves on the Acurian InSite Advisory Board. Fatima Ahmad, Jaime D. Blais, Joseph Chiodo, and Frank S. Czerwiec are employees of Otsuka Pharmaceuticals Development & Commercialization, Inc.
